# The Effect of Symbiotic Ant Colonies on Plant Growth: A Test Using an *Azteca-Cecropia* System

**DOI:** 10.1371/journal.pone.0120351

**Published:** 2015-03-26

**Authors:** Karla N. Oliveira, Phyllis D. Coley, Thomas A. Kursar, Lucas A. Kaminski, Marcelo Z. Moreira, Ricardo I. Campos

**Affiliations:** 1 Programa de Pós-Graduação em Ecologia, Departamento de Biologia Geral, Universidade Federal de Viçosa, Viçosa, Minas Gerais, Brazil; 2 Department of Biology, University of Utah, Salt Lake City, Utah, United States of America; 3 Smithsonian Tropical Research Institute, Panama City, Panama; 4 Institut de Biologia Evolutiva (CSIC-UPF), Passeig Marítim de la Barceloneta, Barcelona, Spain; 5 Laboratório de Ecologia Isotópica, Centro de Energia Nuclear na Agricultura—CENA, Universidade de São Paulo—USP, Piracicaba, São Paulo, Brazil

## Abstract

In studies of ant-plant mutualisms, the role that ants play in increasing the growth rates of their plant partners is potentially a key beneficial service. In the field, we measured the growth of *Cecropia glaziovii* saplings and compared individuals that were naturally colonized by *Azteca muelleri* ants with uncolonized plants in different seasons (wet and dry). We also measured light availability as well as attributes that could be influenced by the presence of *Azteca* colonies, such as herbivory, leaf nutrients (total nitrogen and δ^15^N), and investments in defense (total phenolics and leaf mass per area). We found that colonized plants grew faster than uncolonized plants and experienced a lower level of herbivory in both the wet and dry seasons. Colonized plants had higher nitrogen content than uncolonized plants, although the δ^15^N, light environment, total phenolics and leaf mass per area, did not differ between colonized and uncolonized plants. Since colonized and uncolonized plants did not differ in the direct defenses that we evaluated, yet herbivory was lower in colonized plants, we conclude that biotic defenses were the most effective protection against herbivores in our system. This result supports the hypothesis that protection provided by ants is an important factor promoting plant growth. Since *C*. *glaziovii* is widely distributed among a variety of forests and ecotones, and since we demonstrated a strong relationship with their ant partners, this system can be useful for comparative studies of ant-plant interactions in different habitats. Also, given this study was carried out near the transition to the subtropics, these results help generalize the geographic distribution of this mutualism and may shed light on the persistence of the interactions in the face of climate change.

## Introduction

*Cecropia* (Cecropiaceae) is a genus of fast-growing Neotropical tree, and is an iconic example of ant-plant mutualisms. The majority of *Cecropia* species are myrmecophytes, *i*.*e*. possess specialized structures for housing and feeding symbiotic ants [1]. Although most *Cecropia* plants are inhabited by ants, usually in the genus *Azteca* (Formicidae: Dolichoderinae), seedlings do not begin their lives in association with ants [2]. They first have to develop traits to attract ant partners, such as the hollow stems that provide nesting space and the trichilia, located at the base of each leaf petiole, that produce the glycogen-rich Müllerian bodies that feed ants [2, 3, 4]. In turn, ants defend plants against herbivorous insects [1, 5, 6] and encroaching vines [7], and can also increase nutrient availability for their host plants [8].

Among the benefits provided by symbiotic ants, an increase in plant growth rate might be considered one of the most important [1]. However, the effect of mutualistic ant nests on plant development has received limited study [9], especially for *Cecropia* [5, 10, 11].

Here, we consider three ways by which the presence of *Azteca* might influence the growth of *Cecropia*: by protecting them against herbivory and pathogens [1, 4]; by increasing nutrients in the host plant [8, 12]; and by reducing the necessity for host plant investment in chemical and physical defenses thereby saving energy to be used in growth [13]. These mechanisms are not mutually exclusive, but so far, no studies have evaluated the relevance of these factors at the same time.

Several studies have shown that herbivory can reduce plant growth and fitness by increasing the susceptibility of plants to pathogens vectored by herbivores and the costly loss of tissue [14, 15, 16, 17]. Thus, *Azteca* ants, which are aggressive, could contribute to higher growth rates in colonized plants by decreasing herbivory [18]. Ants could also contribute to plant growth by increasing or recycling nutrients in host plants. Some studies found that nitrogen may flow from ants to *Cecropia* plants since *Azteca* can accumulate organic matter inside the domatia (e.g., debris, storing food), providing external nitrogen sources for the plant [8, 12, 19]. In addition, as an alternative strategy to protection by ants, it has also been suggested that *Cecropia* plants without *Azteca* ants can increase their investment in other defensive strategies, which may also result in slower growth due to greater resource allocation to direct defense [1, 4]. Thus, uncolonized plants not receiving ant protection would not only have to invest in food bodies, but also in direct defenses.

Although the myrmecophytic *Cecropia*-*Azteca* systems are widely known, most studies were conducted with species from Amazonia and Central America (see review [2]). There are few studies in other biomes, such as the Atlantic Forest of Brazil, which has a lower richness of myrmecophytic ant systems than the Amazon Region and Central America [20]. Furthermore, few studies include seasonality, which can affect the outcomes of ant-plant interactions [21]. Such studies can elucidate the generality of the observed patterns for *Cecropia-Azteca* interactions.

In order to investigate if the presence of ant colonies increases plant growth rates and alters herbivory, nitrogen content and defense investments, we focused on *Cecropia glaziovii* Snethl. (Cecropiaceae) that is naturally colonized by *Azteca muelleri* (Emery 1893) ants in an Atlantic Forest remnant in southeastern Brazil. We specifically addressed the following questions: (1) Do plants colonized by *Azteca* ants have higher growth rates than uncolonized plants in wet and dry seasons?; (2) Do colonized plants have lower herbivory rates than uncolonized plants in both seasons?; (3) Do colonized plants have higher nitrogen content than uncolonized plants and is this due to fertilization by ants; and (4) Is there less investment in chemical and physical defenses in colonized than in uncolonized plants?

## Methods

### Study site and system

**Fig 1:**
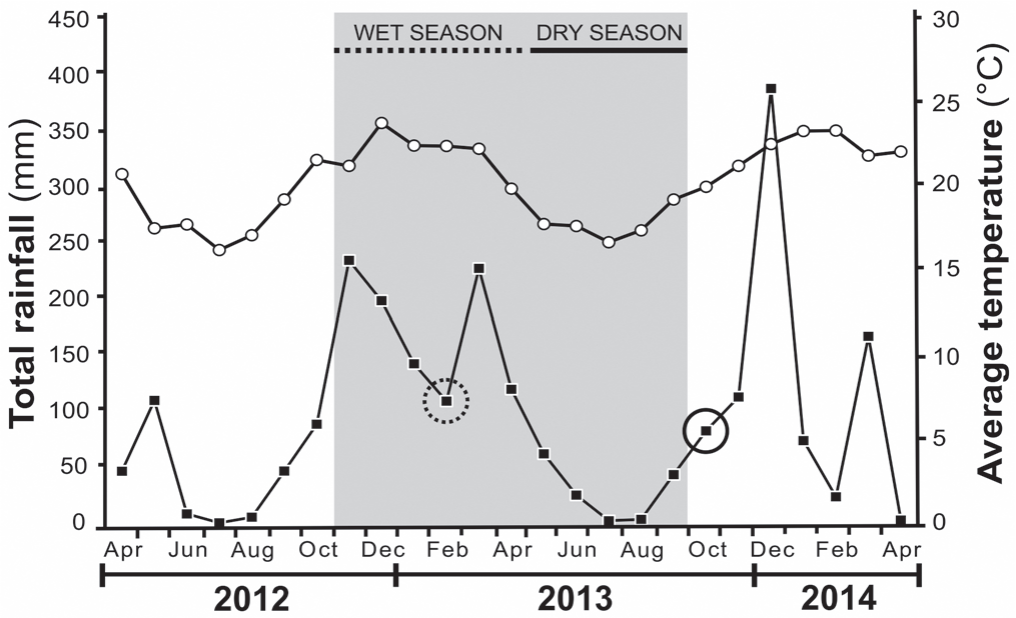
Monthly rainfall and monthly average temperature in the study site. Data obtained from Viçosa Station (INMET) from April 2012 to April 2014. *C*. *glaziovii* growth rate was evaluated during the wet season (the dotted black line at the top of the graph) and the dry season (the continuous black line). The dashed black circle indicates the timing of the herbivory measurement in the wet season, the solid black circle indicates herbivory measurement in the dry season, full squares denote rainfall and open circles denote average temperature.

The study was carried out in the Estação de Pesquisa, Treinamento e Educação Ambiental Mata do Paraíso (EPTEA-MP), Viçosa (20°48’07” S, 42°51’31” W, 648 m asl), Minas Gerais State, Brazil. This area is a private reserve owned by the Federal University of Viçosa (UFV) and all our field sampling was conducted with the permission of the reserve manager Dr. Gumercindo Souza Lima, (Department of Forestry Engineering, UFV). This reserve is a small fragment (195 ha) of the Atlantic Forest of southeastern Brazil characterized by secondary successional forest without disturbance events since 1963. According to the Köopen classification the climate in this area is Cwb, which means that there is a dry and cool winter and a warm summer [22]. The annual rainfall in this area varies from 1,300 to 1,400 mm, and the average annual temperature is 19°C [23], (Fig. 1).

*Cecropia glaziovii* Snethl. (Cecropiaceae) is a common tree of the Atlantic Forest of Brazil, popularly known as ‘embaúba’ [24]. This species reaches up to 20 m in height; the leaves are often large (to ca. 100 x 100 cm). *C*. *glaziovii* is associated with ants and usually occurs in areas of secondary growth or forest borders, widely spread from sea level to ∼1300 m of altitude [24, 25]. In our study area, *C*. *glaziovii* is found naturally in high-light treefall gaps. As noted, *C*. *glaziovii* is colonized by *A*. *muelleri*, an aggressive ant [26], that also is the most important predator of *Coelomera ianio* (Coleoptera: Chrysomelidae), the major defoliating beetle on *Cecropia* spp. [6, 27].

### Plant growth

We selected 48 *C*. *glaziovii* individuals, 25 of which were naturally colonized by *A*. *muelleri* and 23 which were not. All plants were less than 3-m tall (0.38–2.99 m; S1 Fig.) and distributed in sunny areas with similar light availability or canopy openness (S2 Fig.). Plants were carefully inspected for ant presence, which was determined by vigorously shaking the plant stem and subsequently observing the presence/absence of workers in response to the disturbance event. Thus, we considered colonized plants those that had active worker ants. All plants had their height measured during the beginning and end of the rainy season and also in the beginning of the dry season in November 2012, April 2013, and September 2013. In this and other studies, *C*. *glaziovii* growth has been found to be isometric, which means the diameter is directly proportional to stem height [25, 28] (S3 Fig.). For this paper, we quantified the growth rate of *C*. *glaziovii* as the increase in height (cm/day). Growth rate was not related to initial size (S4 Fig.).

Growth rate (cm/day) was calculated for wet and dry seasons using the increment in stem height: GR = (H_final_—H_initial_)/t, where H is height measured from the ground to the apex of the terminal internode, and t is time in days. We also counted the number of mature trichilia (those that were producing Müllerian bodies) per plant. The trichilia pads at the base of the petiole are large and conspicuous, and on mature trichilia, the Mullerian bodies can be seen on the surface or by pressing the leaf petiole towards the plant’s stem.

### Herbivory

To determine whether there were differences in herbivory between colonized and uncolonized plants in the wet season (February 2013), we randomly selected 30 individuals of *C*. *glaziovii* from the same individuals previously evaluated for growth; 15 were naturally colonized by ants and 15 were not. Again, in the end of dry season (October 2013), 39 different individuals were assessed, of which 20 were colonized and 19 uncolonized. For each individual plant, the three most basal leaves were selected and photographed in the field against a white board with 1-cm marks as a reference scale to quantify leaf damage (herbivory). We calculated the total leaf area and the area removed by chewing herbivores using Image J software. The proportion of herbivory was obtained by calculating removed area/total area for each leaf, and then averaged per plant. Since plants were being evaluated for growth rate, removing their leaves to measure herbivory might have influenced their growth. Hence, all the herbivory measurements from February were conducted with the aid of a ladder without removing the leaves. We also noted that some trichilia were infected by a fungus that prevented production of food bodies. We scored the percent of trichilia on all focal plants that were damaged by fungi.

### Nitrogen analysis

To evaluate the nitrogen content and the isotope signature in colonized and uncolonized plants, we selected the same 30 individuals of *C*. *glaziovii* (15 colonized, 15 uncolonized) that we evaluated for herbivory in the dry season. For each individual plant, the three most basal leaves were removed to dry at 40°C for 96 h. Dried plant material was ground to a fine powder, and weighed (2 to 3 mg) in tin capsules for isotope ratio and elemental analyses by Continuous Flow Isotope Ratio Mass Spectrometry, employing a Thermo Scientific Delta Plus mass spectrometer coupled to a Carlo Erba CHN 1110 elemental analyzer (Isotope Ecology Laboratory of the Center for Nuclear Energy in Agriculture, CENA, University of São Paulo). The sample ^15^N/^14^N isotope ratios were compared to an international standard, atmospheric N_2_. Precision, estimated from the reproducibility of the international standards IAEA-N1 and IAEA-N2 [29], was better than ±0.5°/oo (2σ). Results are expressed relative to the standards in "delta" notation:

$$\delta^{15}\text{N }=\left[\left({\text{R}}_{\text{sample}}/{\text{R}}_{\text{standard}}\right)-1\right]*1000,$$

where R is ^15^N/^14^N.

### Chemical and physical defense

In October 2013, we quantified plant investment in physical and chemical defenses as leaf mass per area (LMA) and total phenolics. We removed three leaves from 48 individual plants, of which half were colonized by ants. While still fresh, leaf area was measured and leaves were dried at 40°C for 96 h and weighed to obtain LMA. For total phenolics, the dried leaves were ground to a powder, weighed, and extracted for 1 h at room temperature with 1ml of aqueous methanol (50:50, v/v of water/methanol) per 50 mg of leaf dry weight. We performed three replicate extractions per sample, and the total phenolic content was determined following the Folin-Denis assay [30], using chlorogenic acid as a standard. The average total phenolics for each plant was expressed as milligrams of total phenolics per gram of plant dry mass.

### Data Analysis

To assess the effect of ant colonization and season on plant growth rate, we used the linear mixed effect model (LMER) function of the lme4 package in software R 3.1.0 [31]. We used this analysis as data were obtained repeatedly on the same plants during subsequent intervals (seasons), and this violates the assumption of sampling independence [32]. We also included initial plant height as a covariate to determine whether it affected plant growth (S4 Fig.). We did not include initial stem diameter in this model since *C*. *glaziovii* growth is isometric, and for this reason plant height and stem diameter are correlated (S3 Fig.). The treatment (colonized vs. uncolonized), season, initial height and the interaction between these variables were used as explanatory variables (fixed effects), and as random effects, we assigned the individual plant ID (simulating random blocks) and sampling season (simulating repeated measures in time). We started by fitting a model with all the variables of interest, and in the next model the least significant variable was dropped. When two models were not significantly different, we chose the best-fitting model that had the fewest parameters. Finally, when significant effects of treatment and season were found, we conducted *post-hoc* tests, using the ‘glht’ function in the ‘multcomp’ package [33] for multiple comparisons (S1 Table).

We recorded 5 uncolonized individuals with fungi on the trichilia and none on colonized plants. In order to determine whether fungi in the trichilia influenced plant growth rates, we performed the same growth analysis, using only individuals without fungi. Since the results did not change (S5 Fig.), we reported the growth rate results with all of the individuals evaluated. Finally, to minimize ontogenetic effects in the growth analyses, we also compared the growth rates of 12 colonized and 12 uncolonized plants, with very similar diameters, heights and numbers of leaves (S6 Fig.).

To test the effect of treatment (colonized vs. uncolonized) and season (wet vs. dry) on herbivory, we used generalized linear models (GLM) in R 3.1.0. We also evaluated the effects of treatment on plant traits (δ^15^N signature, total nitrogen content, total phenolics, LMA) using GLMs (S2 Table). The GLM model was compared with null models and minimal adequate models were adjusted with removal of non-significant terms [32]. After residual analysis, the best herbivory model included Gamma errors, and the best models for all other response variables were those with Gaussian errors. The mean proportion of herbivory was the response variable, and treatment, season and interaction between these variables were the explanatory variables.

## Results

**Fig 2:**
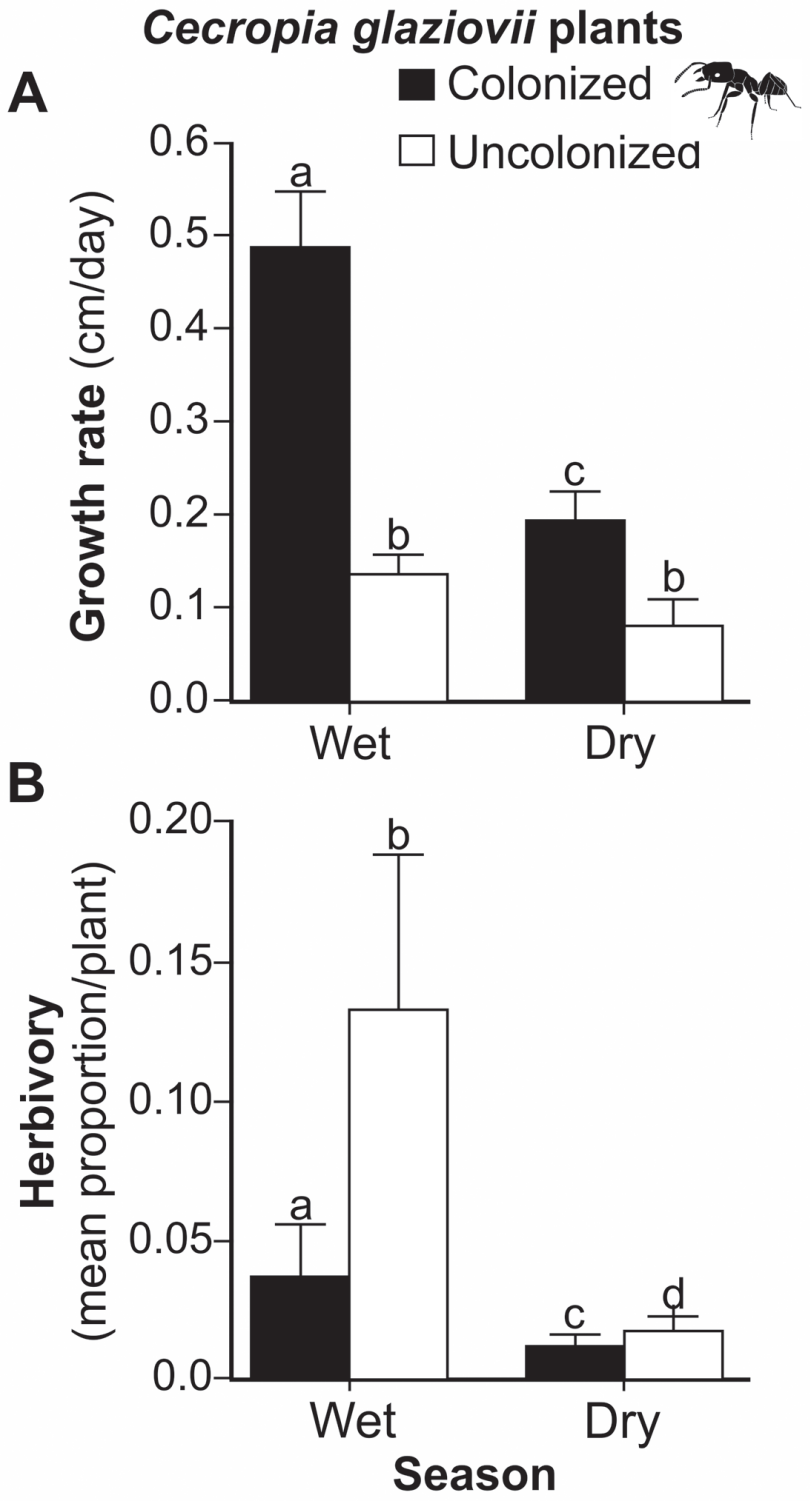
Growth rate (cm/day) (A) and herbivory (B) in wet and dry season. Treatments are *Cecropia glaziovii* plants colonized by *Azteca muelleri* ants (black bars) and uncolonized *C*. *glaziovii* (white bars). Data were shown as mean **±** SE. Different letters above the bars represent statistically different means (P<0.05).

We found that colonized plants grew 3.7 times faster than uncolonized plants and had substantially higher growth rates in the wet as compared to the dry season (χ^2^ = 15.46; *P*<0.01; Fig. 2A). In contrast, there was no seasonal difference in growth rates for uncolonized plants (z = 1.18; *P* = 0.24; Fig. 2A). We found similar results when we performed the statistical analysis with only those individuals of the same size and number of leaves; colonized plants grew faster than uncolonized plants (χ^2^ = 4.15; *P*<0.05; S6 Fig.). Although, initially, colonized plants were slightly taller (F_(1,46)_ = 4.54; *P*<0.05; S2 Fig.), there was no effect of initial height on growth (χ^2^ = 0.03; *P* = 0.87; S5 Fig.). There was also no difference between colonized and uncolonized plants in the proportion of leaves with mature trichilia (S1 Fig.).

**Fig 3:**
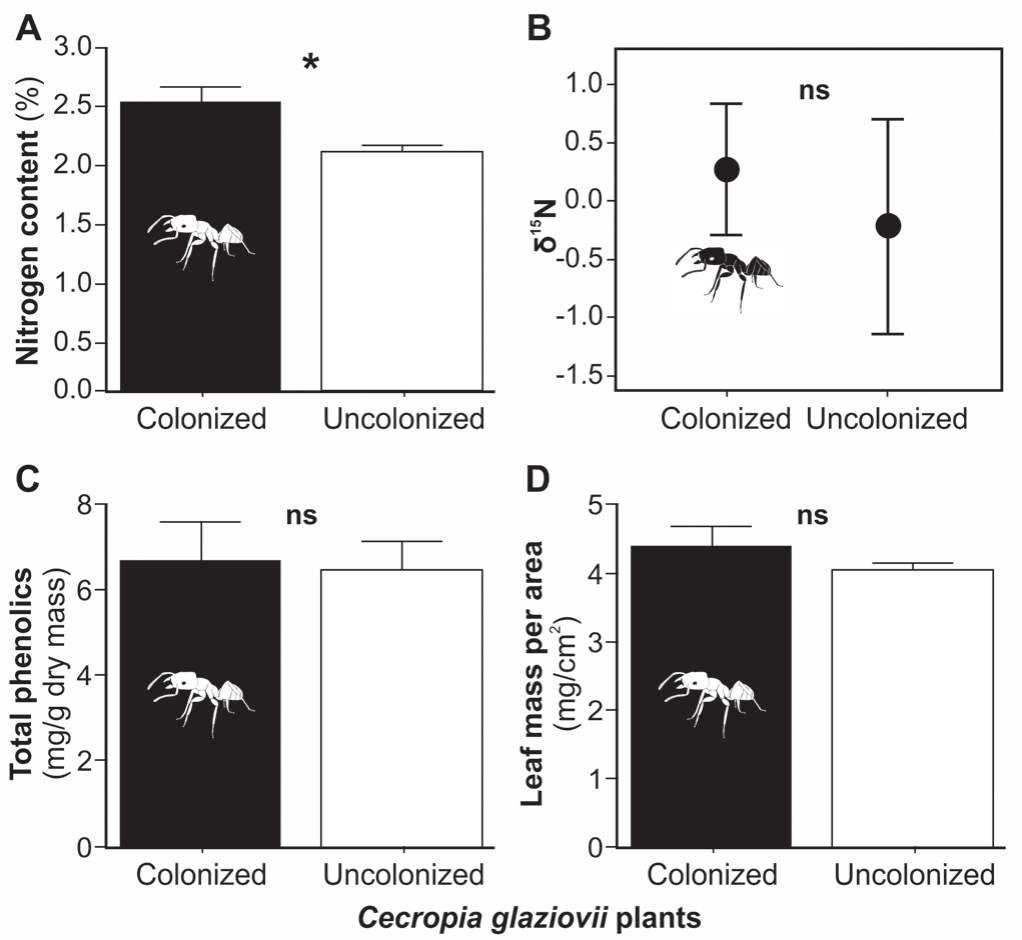
The nitrogen content (A), isotope signature of ^15^N (B), total phenolics (C) and LMA (D). Treatments are *Cecropia glaziovii* plants colonized by *Azteca muelleri* ants (black bars) and uncolonized *C*. *glaziovii* (white bars). Data were shown as mean ± SE. * represents statistically different means (P<0.05).

As predicted, plants colonized by ants experienced less herbivory than uncolonized plants. Moreover, the same effect of ants was found in both seasons (F_(1,67)_ = 7.8; *P*<0.01, Fig. 2B) and the herbivory rate was higher in the wet season for both treatments (F_(1,66)_ = 16.39; *P*<0.001; Fig. 2B). As we hypothesized, colonized plants had a higher nitrogen content than uncolonized plants (F_(1,28)_ = 8.51; *P*<0.01; Fig. 3A), although the isotopic signature, an indicator of the trophic source of nitrogen, did not differ between colonized and uncolonized plants (F_(1,28)_ = 0.96; *P* = 0.34; Fig. 3B). Also, we detected no difference between treatments in total phenolics (F_(1,46)_ = 0.04; *P* = 0.85; Fig. 3C), leaf mass per area (LMA) (F_(1,46)_ = 1.47; *P* = 0.23; Fig. 3D) or light environment (F_(1,41)_ = 2.38; P = 0.13; S2 Fig.).

## Discussion

Colonized plants grew faster than uncolonized plants in both seasons, and this result is independent of *C*. *glaziovii* ontogeny. Considering all results together, we suggest that the most likely explanation for enhanced growth is the presence of ants. However since we did not experimentally manipulate the presence of ants, we cannot definitively determine whether the differences in plant performance were because the presence of ants caused plants to grow larger or because colonized plants were healthier prior to colonization. Nonetheless, even if ant colonies preferentially establish on healthier plants, colonized *Cecropia* would still receive the protection from herbivores that clearly improves growth. Our results corroborate other studies showing that ants protect myrmecophytic plants against herbivory [4, 5, 9, 34, 35]. Silveira et al. [27] found that *A*. *muelleri* effectively defended *Cecropia* against a specialist herbivore, *Coelomera ianio* (Coleoptera: Chrysomelidae). In fact, we found this beetle on some uncolonized plants in our study area (K. Oliveira, pers. obs.). It is probable that the lower herbivory found in our study for colonized plants is due to the aggressive behavior of *A*. *muelleri* [6], and this could contribute to higher plant growth rates. Poorter [36], in a study with six genera of plants, including *Cecropia*, found that a 10 percent reduction in leaf area leads to a 10 percent reduction in height growth for saplings. Similarly, Marquis [37] found, for a tropical shrub, *Piper arieianum*, that removal of 10 percent of leaf area from single reproductive branch produced 80% fewer viable seeds, and those damaged branches grew less, thus affecting plant fitness.

The highest herbivory rate recorded in this study was for uncolonized plants in the wet season (13.3% ± 0.05), which is similar to other studies from tropical forests [17, 38, 39]. For *Cecropia* spp., Coley [40] found 12–18% of herbivory, while in other myrmecophytes the herbivory rate varied between 4–12% [18]. In our study, the herbivory rate was higher in the wet season, as found for *Cecropia insignis* [40] and *Cecropia peltata* [4]. These results might be due to an increase in the populations of herbivores in this season. Insects change in abundance with seasonality, especially where wet and dry seasons alternate [41, 42]. In the wet season, new leaves are produced and herbivorous insect populations synchronize their cycles with food availability, leading to greater rates of herbivory [17, 41]. The fact that the differences between colonized vs. uncolonized plants in growth rate and herbivory were much greater in the wet season has implications for the strength of the mutualism in the face of climate change. Since this region is likely to experience more extreme or longer dry seasons under climate change [43], and consequently lower herbivore pressure, the strength of the mutualism may weaken in the future [44].

It is well known that the production of different defensive strategies has high energetic costs for plants, especially in limited resource environments [13, 45, 46]. Moreover, once a resource is used in chemical defense production, it cannot be used in other processes such as plant growth or reproduction [45, 47]. Thus, based on the fact that colonized plants already invest in food body production for ant protection [48, 49, 50], we can suppose that it will be unnecessary for them to invest in other defensive strategies, and thus plants could grow faster. Here, we found that colonized and uncolonized plants had the same proportion of functional trichilia, showing that both groups produced Müllerian bodies. Even if uncolonized plants produce fewer food bodies than colonized plants [11, 50], uncolonized plants still pay a cost but receive no benefit from ants. This cost without a benefit could negatively impact the growth of uncolonized plants. Our results do not support the hypothesis that the investment in chemical and physical defenses is higher in uncolonized individuals, or that colonized plants reduce their chemical defense in order to avoid 'superfluous' costs resulting from redundant defenses. We conclude that ants, rather than chemical or mechanical defense, are responsible for the lower herbivory in colonized plants. Phenolics, the most widespread and abundant class of secondary metabolites, and LMA, also considered to be effective against herbivores [17, 40, 51], did not differ between treatments. Although we did not quantify all classes of secondary metabolites, our results suggest that uncolonized plants make similar investments in direct defense as colonized plants. The maintenance of direct defenses in colonized plants may be because not all herbivores are deterred by ants [52, 53]. Moreover, phenolic compounds, particularly flavonoids and anthocyanins, can protect against photodamage [54].

Another mechanism by which ants can increase plant growth is through nitrogen supplementation. Although we found a difference in nitrogen content between colonized and uncolonized, this result cannot be attributed to the trophic upgrading that would be expected from ant-derived nutrients since we did not find a difference in isotope signature. If ants supply their host with added nutrients, then such plants should be richer in ^15^N than those without ants [8]. We found a non-significant trend for higher δ^15^N in colonized versus uncolonized plants in our study. Defossez et al. [55] suggested that for tropical trees, nitrogen is not the best nutrient on which to focus because it is not as limiting as it is for ant–epiphyte symbioses described in the first studies of nutrient flux from ants to plants. Fisher *et al*. [56] also found that ant-derived nitrogen has minor importance for the ground-rooted understory myrmecophytes. In our study we worked with relatively young plants (ranging from 0.38 to 2.99 m height), and it may be that the soil is a sufficient source of N. Recently it has been suggested that fungal partners could improve nitrogen transfer from ants to plants, since complex molecules, such as ant debris, may not be absorbed by the surface of domatia [57, 58]. However, this remains unevaluated for the *Azteca-Cecropia* system. We also found that colonized plants had higher total nitrogen content than uncolonized, but we cannot relate it directly to ant fertilization since, as noted, we did not find a significant difference in isotope signatures.

In addition to the effect of herbivory, defensive trade-offs, and nitrogen on *Cecropia* growth rates, it has been hypothesized that *Azteca* ants could benefit the plants by providing parasite control. In a *Cecropia-Azteca* system in French Guiana, plants with *Azteca* ants had a lower percentage of trichilia infected by the fungus *Fusarium moniliforme* and higher height than uncolonized plants [11]. This fungus produces growth-inhibiting mycotoxins that cause necrosis in plants [59]. Roux et al. [11] suggested that ants protected plants against the fungus attacking trichilia, which, in turn, might improve plant growth. In our study we found only five individuals with visible fungus on trichilia, and the growth analysis excluding them did not change our results. Thus, although we cannot totally discard the role of pathogens in plant growth, they did not influence our results.

Although we think it likely that ants are the main reason for enhanced growth, another possibility is that colonized plants were healthier prior to colonization and queens preferentially chose or survived better in healthier plants. Higher light conditions would be an obvious cue for queens, but light regimes were the same for colonized and uncolonized plants (S2 Fig.). Of all the leaf traits we measured, only foliar nitrogen content differed between colonized and uncolonized plants. Higher nitrogen could lead to greater photosynthesis and growth but is unknown whether *Azteca* queens in flight can use leaf N as an indication of a healthy host. Additionally, healthier plants may favor later stages of establishment. After landing on the chosen host, the founding queen recognizes the prostomata area, gnaws an entrance hole, enters the stem, and establishes colonies in the domatia [49, 60]. The interactions occurring inside the domatia that affect establishment are also unknown, but it is common in *Cecropia* to find several dead and moribund *Azteca* queens inside the basal internodes [61]. Perhaps, after colonization, ant queens may be more successful in colony establishment in healthier host plants. Thus, we cannot rule out the possibility that healthier plants grow faster and also promote the establishment of ant colonies.

Ecological and evolutionary benefits and costs of ant-plant mutualism may vary among species pairs, plant age, local environmental conditions and identity and the abundance of ant partners [19, 21, 62]. We show here that *Cecropia* plants colonized by *Azteca* colonies have higher growth rates than plants without ants, and that this is most likely related to protection against herbivory and perhaps higher nitrogen content. Our results show similar patterns in both seasons which suggest a strong mutualistic relationship between *C*. *glaziovii* and *A*. *muelleri* even in the face of environmental fluctuations [21]. Moreover, our patterns are similar to studies of *Cecropia* in other environments, such as the Amazon and Central America. Since *C*. *glaziovii* is widely distributed among a variety of forests as well as at different elevations in southern Brazil, and since we demonstrated a strong relationship with their ant partners, this system can be useful for comparative studies of ant-plant interactions in different habitats, and may help predict the persistence of the interaction in the face of climate change [46]. Finally, studies that focus on ant colony establishment and the frequency of plant occupancy in different altitudinal and spatial scales along the geographic distribution of *C*. *glaziovii* seem a promising topic for future research.

## Supporting Information

S1 Fig Initial traits of the colonized and uncolonized individuals by ants. (DOC)

S2 Fig Canopy openness for plants colonized by ants vs. plants that are uncolonized. (DOC)

S3 Fig The relationship between initial height and stem diameter in *Cecropia glaziovii*. (DOC)

S4 Fig The relationship between growth rate and initial height for both treatments. (DOC)

S5 Fig Growth rate (cm/day) for plants without fungus attack to trichilia. (DOC)

S6 Fig Growth rate (cm/day) for plants with the same height and diameter. (DOC)

S1 Table Linear mixed effects models for *C*. *glaziovii* growth. (DOC)

S2 Table Analyses of deviance of the models showing the effects of traits in *C*. *glaziovii*. (DOC)
