# Supplementary material for: The Effect of Symbiotic Ant Colonies on Plant Growth: A Test Using an Azteca-Cecropia System
Source: PLoS One. 2015 Mar 26;10(3):e0120351. doi: 10.1371/journal.pone.0120351 (PMC4374854; doi:10.1371/journal.pone.0120351)
Supplement: S1 Fig — (DOC) [file pone.0120351.s001.doc]

**S1 Fig. Initial traits of individuals of colonized by ants vs. those that were not colonized.** At the beginning of this study, colonized individuals had heights between 0.38 to 2.99 m and 3 to 12 leaves (8.33 ±0.69; mean ± SE), while uncolonized individuals varied between 1.10 to 2.08 m with 3 to 8 leaves (4.84 ±0.30). There was a significant difference in initial height between colonized plants (black bars) and uncolonized plants (white bars) (F_(1,46)_= 4.54; *P*<0.05) and in diameter stem (F_(1,38)_=22.30; *P*<0.001). There was no difference in the proportion of leaves with Müllerian bodies (MBs) between colonized and uncolonized plants (F_(1,38)_=1.50; *P*=0.23). Different letters above the bars represent statistically different means (P<0.05).
