# Supplementary material for: The Effect of Symbiotic Ant Colonies on Plant Growth: A Test Using an Azteca-Cecropia System
Source: PLoS One. 2015 Mar 26;10(3):e0120351. doi: 10.1371/journal.pone.0120351 (PMC4374854; doi:10.1371/journal.pone.0120351)
Supplement: S2 Fig — (DOC) [file pone.0120351.s002.doc]

**S2 Fig. Canopy openness for plants colonized by ants vs. plants that were uncolonized.** In July 2014, in order to confirm that colonized and uncolonized plants had the same light availability, we selected 22 colonized and 21 uncolonized individuals that were also evaluated for growth. The other five plants from our growth evaluation were dead. Hemispherical photographs were taken under each plant, at a standardized height of 1m from soil. These photos were taken around sunrise when the sky was overcast. Photos were taken with an Fc-E9 fisheye adapter lens mounted on a Nikon Coolpix 5700 camera. The images were analyzed in Gap Light Analyzer software [1], and we used the percentage canopy openness to estimate light-availability. There was no difference between the canopy openness for colonized or uncolonized plants (F_(1,41)_=2.38; *P*=0.13). There was also no effect of light availability on plant growth (F_(1,41)_=0.43; *P*=0.51). **Growth rate was calculated for the entire period of study (Height (September 2013) – Height (November 2012) / 249 days) in order to verify the effect of light on plant growth.

**Reference**

1. Frazer GW, Canham CD, Lertzman KP (1999) Gap Light Analyzer (GLA), Version 2.0: Imaging software to extract canopy structure and gap light transmission indices from true-colour fisheyephotographs, users manual and program documentation. New York: Simon Fraser University, Burnaby, British Columbia, and the Institute of Ecosystem Studies, Millbrook.
