# Supplementary material for: The Effect of Symbiotic Ant Colonies on Plant Growth: A Test Using an Azteca-Cecropia System
Source: PLoS One. 2015 Mar 26;10(3):e0120351. doi: 10.1371/journal.pone.0120351 (PMC4374854; doi:10.1371/journal.pone.0120351)
Supplement: S3 Fig — (DOC) [file pone.0120351.s003.doc]

**S3 Fig. The relationship between initial height and stem diameter in *C. glaziovii*.** a) all plants (F_(1,38)_=5.44, *P*<0.05), and b) the same relationship for colonized (solid line) and uncolonized plants (dashed line) separately (F_(1,38)_=12.66, *P*<0.01).We used generalized linear models (GLMs) with a Gamma error structure.
