# Supplementary material for: The Effect of Symbiotic Ant Colonies on Plant Growth: A Test Using an Azteca-Cecropia System
Source: PLoS One. 2015 Mar 26;10(3):e0120351. doi: 10.1371/journal.pone.0120351 (PMC4374854; doi:10.1371/journal.pone.0120351)
Supplement: S4 Fig — (DOC) [file pone.0120351.s004.doc]

**S4 Fig. The relationship between growth rate and initial height for both treatments.**

Open circles represent uncolonized individuals and solid circles represent colonized individuals in (a) wet season and in (b) dry season. There was no effect of initial height on growth rate (χ^2^= 0.027; P=0.867).
