# Supplementary material for: The Effect of Symbiotic Ant Colonies on Plant Growth: A Test Using an Azteca-Cecropia System
Source: PLoS One. 2015 Mar 26;10(3):e0120351. doi: 10.1371/journal.pone.0120351 (PMC4374854; doi:10.1371/journal.pone.0120351)
Supplement: S5 Fig — (DOC) [file pone.0120351.s005.doc]

**S5 Fig. Growth rate (cm/day) for plants without fungus attack to trichilia.** Treatments are plants colonized by ants (black bars) and uncolonized (white bars). Different letters above the bars represent statistically different means (P<0.05). Colonized plants grew faster than uncolonized plants and had higher growth rates in the wet as compared to the dry season (χ^2^=13.86; *P*<0.01). In contrast, there was no seasonal difference in growth rates for uncolonized plants (z =0.97; *P*=0.33).
