# Supplementary material for: The Effect of Symbiotic Ant Colonies on Plant Growth: A Test Using an Azteca-Cecropia System
Source: PLoS One. 2015 Mar 26;10(3):e0120351. doi: 10.1371/journal.pone.0120351 (PMC4374854; doi:10.1371/journal.pone.0120351)
Supplement: S6 Fig — (DOC) [file pone.0120351.s006.doc]

**S6 Fig. Growth rate (cm/day) for plants with the same height and diameter.** The treatments are plants colonized by ants (black bars) and uncolonized (white bars). The 12 plants in each treatment had the same height (F_(1,22)_=2.77; P=0.11), diameter (F_(1,22)_= 3.58; P=0.07) and number of leaves (Deviance _(1,22)_= 1.1834; P=0.28). Different letters above the bars represent statistically different means for within-season comparisons. Colonized plants grew faster than uncolonized plants (χ^2^=4.15; *P*<0.05); and had higher growth rates in the wet as compared to the dry season (χ^2^=48.43; *P*<0.001).
