# Supplementary material for: The Effect of Symbiotic Ant Colonies on Plant Growth: A Test Using an Azteca-Cecropia System
Source: PLoS One. 2015 Mar 26;10(3):e0120351. doi: 10.1371/journal.pone.0120351 (PMC4374854; doi:10.1371/journal.pone.0120351)
Supplement: S1 Table — (DOC) [file pone.0120351.s007.doc]

**S1 Table. Linear mixed effects models for *C. glaziovii* growth.**

| Models | **AIC** | **Deviance** | **χ^2^** | **P** |
| --- | --- | --- | --- | --- |
| m1=Ant+Season+InitialHeight**+Ant:Season** | -36.355 | -54.355 | 8.0307 | 0.0046* |
| m2=Ant+Season+InitialHeight | -30.324 | -46.324 | - | - |
| In this step, we choose the model m1 as the best model. | | | | |
| m1=Ant+Season**+InitialHeight**+Ant:Season | -36.355 | -54.355 | 0.0279 | 0.8674 |
| m3=Ant+Season+Ant:Season | -38.327 | -54.327 | - | - |
| Finally, we choose the model m3 as the best model. | | | | |

Bold types were those variables that we tried to remove in the subsequent model. If they were significant different (P<0.05*), they were maintained in the model. Akaike information criterion (AIC) values, Deviance, χ^2^ and P-values are presented for each model. Ant refers to colonized and uncolonized plants, while Season refers to wet and dry season.

**Simultaneous Tests for General Linear H**ypotheses -Multiple Comparisons of Means: User-defined Contrasts

|  | **Estimate** | **Std. Error** | **z value** | **Pr(>|z|)** |
| --- | --- | --- | --- | --- |
| **Col.Wet - Col.Dry** | 0.29606 | 0.04609 | 6.424 | 1.33e-10 * |
| **Uncol. Wet – Uncol.Dry** | 0.05656 | 0.04805 | 1.177 | 0.239 |
| **Uncol. Wet – Col. Wet** | -0.35259 | 0.05335 | -6.609 | 3.86e-11 * |
| **Uncol. Dry – Col. Dry** | -0.11309 | 0.05335 | -2.120 | 0.034 * |

Col means colonized plants, while Uncol means uncolonized plants.
