# Supplementary material for: The Effect of Symbiotic Ant Colonies on Plant Growth: A Test Using an Azteca-Cecropia System
Source: PLoS One. 2015 Mar 26;10(3):e0120351. doi: 10.1371/journal.pone.0120351 (PMC4374854; doi:10.1371/journal.pone.0120351)
Supplement: S2 Table — (DOC) [file pone.0120351.s008.doc]

**S2 Table. Analyses of deviance of the models showing the effects of traits in *C. glaziovii*.** The explanatory variable named Ant represents uncolonized and colonized individuals. Different samples sizes were used for each analysis (See the methodology section for details).

| **Response**  **variable** | **Explanatory**  **variable** | **Error**  **distribution** | **GL** | **Deviance** | **Residual**  **GL** | **Residual**  **Deviance** | **F** | **P** |
| --- | --- | --- | --- | --- | --- | --- | --- | --- |
| Initial height | Null | Gaussian | - | - | 47 | 17.423 | - | - |
|  | Ant | Gaussian | 1 | 1.5651 | 46 | 15.858 | 4.54 | 0.03849* |
| Diameter of stem | Null | Gaussian | - | - | 39 | 3691.1 | - | - |
|  | Ant | Gaussian | 1 | 1427.6 | 38 | 2263.5 | 23.967 | 0.00002* |
| Herbivory | Null | Gamma | 1 | - | 68 | 213.72 | - | - |
|  | Ant | Gamma | 1 | 20.08 | 67 | 193.64 | 7.7958 | 0.0068* |
|  | Season | Gamma | 1 | 42.22 | 66 | 151.42 | 16.392 | 0.0001* |
| Nitrogen content | Null | Gaussian | - | - | 29 | 6.0374 | - | - |
|  | Ant | Gaussian | 1 | 1.407 | 28 | 4.6302 | 8.5085 | 0.00689* |
| δ^15^N | Null | Gaussian | - | - | 29 | 54.91 | - | - |
|  | Ant | Gaussian | 1 | 1.8167 | 28 | 53.093 | 0.9581 | 0.3361 |
| Total phenolics | Null | Gaussian | - | - | 47 | 661.24 | - | - |
|  | Ant | Gaussian | 1 | 0.6219 | 46 | 660.62 | 0.0433 | 0.8361 |
| Leaf mass per area | Null | Gaussian | - | - | 47 | 0.0001 | - | - |
|  | Ant | Gaussian | 1 | 0.000003 | 46 | 0.0001 | 1.4679 | 0.2319 |
